# Surface-Modified Piezoelectric Copolymer Poly(vinylidene fluoride–trifluoroethylene) Supporting Physiological Extracellular Matrixes to Enhance Mesenchymal Stem Cell Adhesion for Nanoscale Mechanical Stimulation

**DOI:** 10.1021/acsami.3c05128

**Published:** 2023-09-18

**Authors:** Hannah Donnelly, Mark R. Sprott, Anup Poudel, Paul Campsie, Peter Childs, Stuart Reid, Manuel Salmerón-Sánchez, Manus Biggs, Matthew J. Dalby

**Affiliations:** ‡ Centre for the Cellular Microenvironment, University of Glasgow, Glasgow G12 8QQ, United Kingdom; § Centre for Research in Medical Devices (CÚRAM), National University of Ireland Galway, Galway H91W2TY, Ireland; ⊥ SUPA Department of Biomedical Engineering, University of Strathclyde, Glasgow G1 1QE, United Kingdom

**Keywords:** tissue engineering, piezoelectric, polymers, mesenchymal stem cells, fibronectin, cell adhesion, osteogenesis

## Abstract

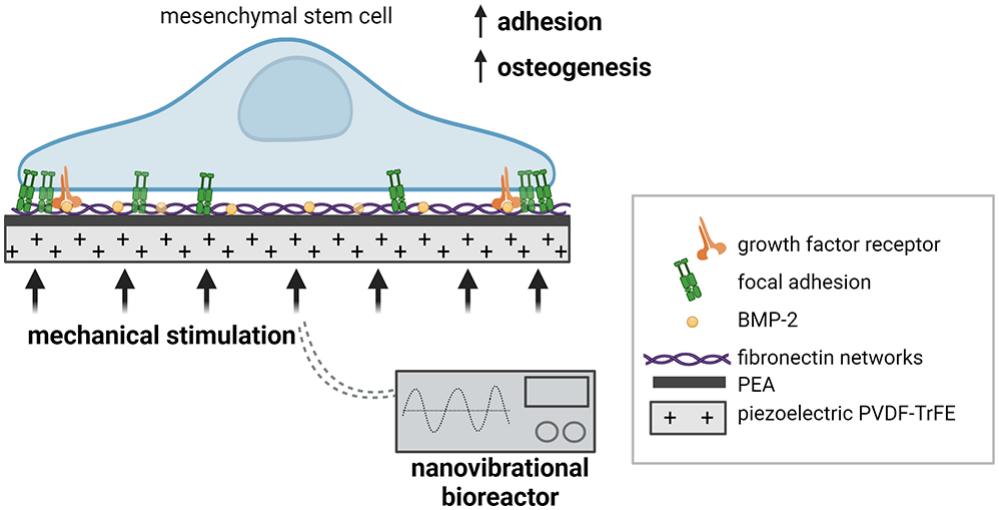

There is an unmet clinical need to provide viable bone grafts for clinical use. Autologous bone, one of the most commonly transplanted tissues, is often used but is associated with donor site morbidity. Tissue engineering strategies to differentiate an autologous cell source, such as mesenchymal stromal cells (MSCs), into a potential bone-graft material could help to fulfill clinical demand. However, osteogenesis of MSCs can typically require long culture periods that are impractical in a clinical setting and can lead to significant cost. Investigation into strategies that optimize cell production is essential. Here, we use the piezoelectric copolymer poly(vinylidene fluoride–trifluoroethylene) (PVDF-TrFE), functionalized with a poly(ethyl acrylate) (PEA) coating that drives fibronectin network formation, to enhance MSC adhesion and to present growth factors in the solid phase. Dynamic electrical cues are then incorporated, via a nanovibrational bioreactor, and the MSC response to electromechanical stimulation is investigated.

## 1 Introduction

With an aging population, understanding how to maintain the musculoskeletal system is important for better quality of life.^1^ However, bone regeneration continues to be challenging in the clinic.^1^ Autologous bone grafts are the gold standard graft material, possessing desirable properties to promote bone repair (osteoinductivity, osteoconductivity, and osteogeneity).^2^ However, its supply is limited, and a second surgical procedure is required to harvest graft material, which is associated with donor site morbidity.^3^ The most common bone-graft substitutes, therefore, are decellularized grafts, yet this method is also limited by donor quality and the risk of host immunogenic responses.^1,3^ This has motivated the development of bone-graft substitutes such as regenerative biomaterials.^4^

Many tissue engineering strategies have been investigated, e.g., synthetic ceramics,^5^ functionalized titanium,^6^ and natural/synthetic polymers.^7,8^ Recently, piezoelectric polymers, materials that develop a voltage when a mechanical stress is applied, have been investigated as potential regenerative biomaterials.^9,10^ Native bone is a piezoelectric substance^11^ that can generate electrical potentials that may influence remodelling and metabolism in regeneration;^12^ this makes piezoelectric materials an interesting candidate for orthopedic applications.

Recently, piezoelectric polymers and their composites have garnered special interest as active scaffolds for tissue engineering applications due to their facile processability, flexibility, biocompatibility, and enhanced cell functionality.^10,13−16^ Piezoelectric poly(vinylidene fluoride) (PVDF) has been investigated for a range of tissue engineering applications.^9^ Indeed, cells have been shown to be sensitive to the presence of different PVDF surface properties such as roughness, chemistry, and surface free energy.^10,13,14,17^ PVDF has been previously investigated for orthopedic applications, and a culture of preosteoblasts on PVDF films in two crystal phases, nonpolar α-PVDF and electroactive β-PVDF, demonstrated differences in cell proliferation in response to polarity.^13^ A copolymer of PVDF and trifluoroethylene (PVDF-TrFE) was recently investigated for tendon repair applications.^10^

In stressed bone, the generation of electrical potentials is dependent on its mechanical deformation.^18^ Recently, a method of applying nanoscale mechanical force to cells was shown as a viable approach to stimulating osteogenesis of mesenchymal stromal cells (MSCs).^19,20^ Using a nanovibrational bioreactor (Nanokick bioreactor), MSCs undergo robust osteogenic commitment in 2D and 3D collagen gels.^19,20^ Nanokicking of MSCs typically requires >28 days of stimulation in order to drive osteogenesis.^21^ In a clinical setting, this turnaround time is impractical^1^ and would require extensive equipment hours at significant cost, limiting throughput as well as longer culture times associated with infection and cell phenotypic drift and viability. Therefore, investigation into cell culture strategies that optimize the Nanokick bioreactor is essential. As such, in this study, we sought to apply mechanical forces from the nanovibrational (or nanokicking, NK) bioreactor to the piezoelectric material PVDF-TrFE, with an aim of incorporating dynamic electrical cues to investigate its potential as a platform to promote osteogenesis of MSCs.

To enhance the functionality of the PVDF-TrFE surface, we coated it with a nanoscale layer (∼10 nm) of poly(ethyl acrylate) (PEA).^22^ PEA has been well characterized as a functional coating for several tissue engineering applications, including driving osteogenesis of MSCs in vitro and bone regeneration in vivo. Typically, when the extracellular matrix (ECM) protein fibronectin (FN) is adsorbed to material surfaces, it adopts a closed, globular conformation, concealing cryptic cell adhesion and growth factor (GF) binding domains. However, PEA drives FN network formation, a process that is usually cell-driven.^23^ This leads to the unfolding of FN molecules and exposes key binding domains, such as the integrin binding RGD and PSHRN synergy sites, and a GF binding domain capable of sequestering and presenting GFs to cells.^24−27^ We therefore coated PVDF-TrFE membranes with PEA to drive FN network formation with the aim of enhancing cell–material interactions and driving MSC adhesion. Then by adsorbing bone morphogenetic protein 2 (BMP-2) to FN to present the GF in the solid phase, we investigate the systems’ future potential to support MSC osteogenesis.

Here, we aim to introduce piezoelectric stimulation via PVDF-TrFE to the NK bioreactor system. PEA and FN coatings are further incorporated to enhance cell adhesion, with further potential to present osteogenic GFs to cells in the solid phase. By incorporating multiple stimuli native to bone, we aim to investigate a system with the potential to promote the osteogenesis of MSCs. PVDF-TrFE is a facile material capable of producing physiologically relevant piezoelectric cues.^10^ It is envisioned that strategies such as this that enhance cell–material interactions could improve the potential of the NK bioreactor to efficiently produce cells of clinical value.

## 2 Materials and Methods

### 2.1 PVDF-TrFE Synthesis and Polarization

The PVDF-TrFE (70–30 mol %, MW = 300 kDa) copolymer was purchased from Solvay in powder form. PVDF-TrFE was dissolved in a 1:1 ratio of a *N*,*N*-dimethylformamide/acetone solution (5:1) and cast onto a glass plate to form films of 20–30 μm thickness. Films were then annealed at 120 °C for 12 h. Electrical pooling was then carried out using a direct-current voltage. Thin films were clamped between two steel electrodes and subjected to an electric field of 100 V/μm for 5 min.

### 2.2 Scanning Interferometry

The noncontact measurements were taken using a Polytec PSV-500H scanning vibrometer (Polytec GmbH, Waldbronn, Germany) under the following conditions: measuring laser, HeNe, 633 nm; sampling frequency, 5.12 MHz; bandwidth, 2 MHz; maximum fast Fourier transform (FFT) lines, 819200; resolution, 195.3 ns. Cultureware was magnetically attached to the top plate, and the laser scanning head of the Polytec system was set up on a tripod that was positioned on an optical bench. Using Polytec’s PSV software, a measurement grid was created across the surfaces of interest, generating laser-based geometry measurements of the *X*, *Y*, and *Z* coordinates of each measurement point in the process (Figure S3). For scans performed at a specific frequency, a sinusoidal waveform voltage signal, generated by a Polytec DAQ system, is sent to a Behringer KM-750 (Behringer, Willich, Germany) audio amplifier to produce the power required to drive the piezoarray of the bioreactor. The drive signal from the amplifier is also routed back to the Polytec DAQ system and used as a reference signal, and trigger, for the measurements. For scans where a model analysis is performed, white noise is generated by a DAQ system, exciting the mechanical structure across a 1000 Hz bandwidth. The FFT settings for the measurements were as follows: FFT lines, 12800; sample time, 1.28 s; sample frequency, 25.6 kHz. Scans of >100 measurement points were taken across the NK bioreactor top plate and of PVDF-TrFE mounted on volcano culture plates at different frequencies and are summarized in Tables S1 and S2.

### 2.3 PEA Plasma Polymerization

The plasma equipment was set up according to our previous work.^22,25,27^ A custom-built plasma reactor was used to polymerize ethyl acrylate (EA) via plasma polymerization. The EA plasma was generated by two capacitively coupled copper band electrodes, which were connected to a radio-frequency (RF) power supply. Details of other design and operation considerations to facilitate the polymerization of EA can be found in our previous work.^22,25^ Briefly, PVDF-TrFE membranes were placed in the plasma chamber vertically to the plasma flow. Then, samples were exposed to air plasma for 5 min at 50 W of RF incident power to ensure the removal of any residual organic matter. For PEA plasma, the RF power applied to the plasma chamber was set as 50 W and the plasma treatment was 15 min. Before the ECM coating for cell culture, the samples were sterilized under ultraviolet light for 30 min.

### 2.4 Mounting PVDF-TrFE Films on Custom Cell Culture Plates

**Figure 1:**
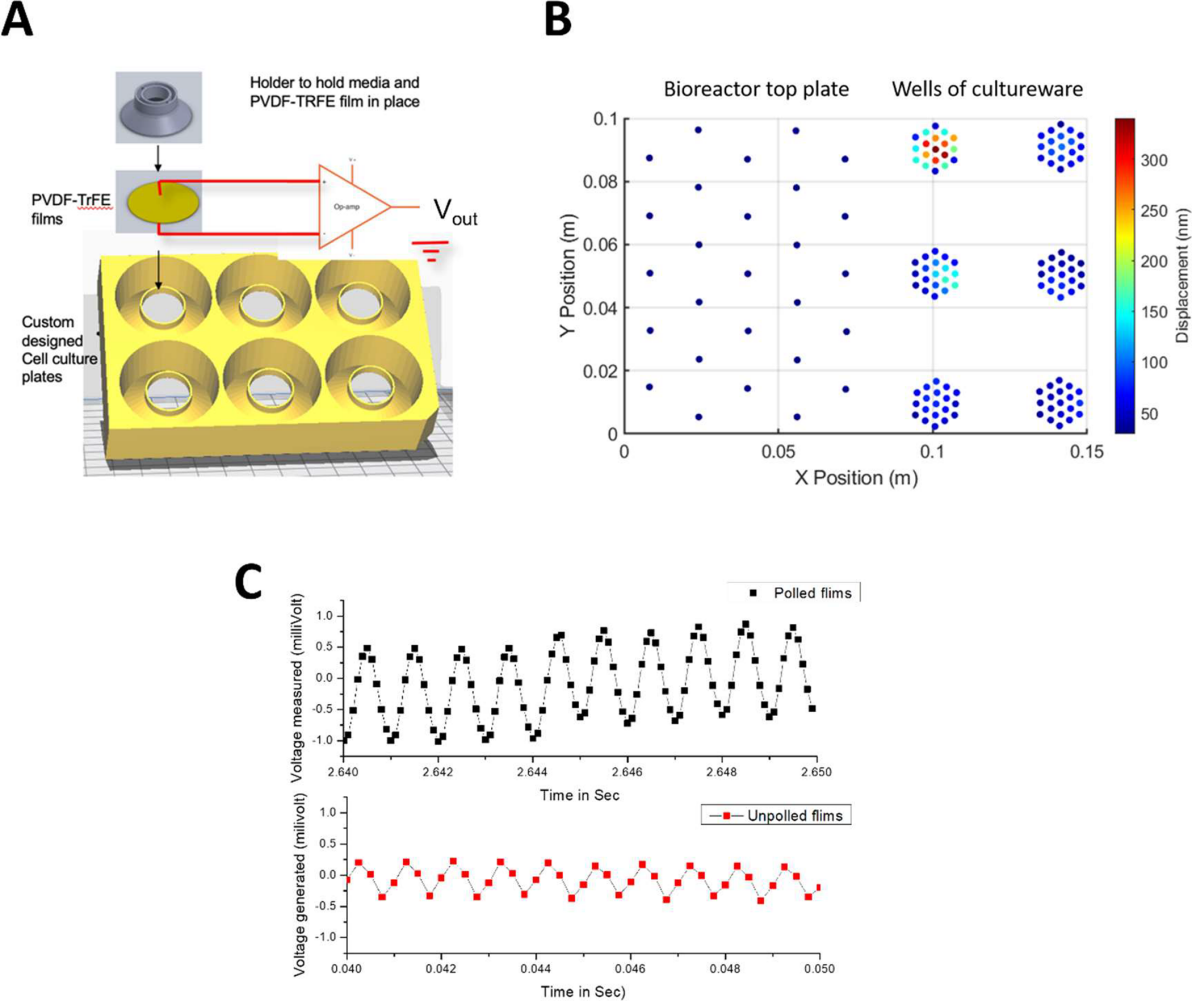
PVDF-TrFE film setup. (A) Schematic of the electromechanical bioreactor culture plate. Thin PVDF-TrFE films were mounted within the cell culture plates and subjected to 1 kHz nanoamplitude vibration. (B) Plot showing the *z*-displacement across a section of the bioreactor top plate and each of the wells of the cultureware. (C) Voltage output of poled and unpoled PVDF-TrFE mounted films at 1 kHz.

Custom cell culture plates were produced by injection molding as previously described.^10^ Culture plates were in a 6-well format and contained a volcano-like shape, as illustrated in Figure 1A, to allow films to be fixed taught in each well. Glue was added to the rim of each volcano, and films were laid across and held in position using custom 3D-printed rings until the glue had set. The rings were then removed, leaving tightly fixed films, and wells were washed three times with phosphate-buffered saline (PBS) before cell seeding.

### 2.5 Differential Scanning Calorimetry (DSC)

DSC was used to investigate the thermal properties of PVDF-TrFE using a TA Instruments 2000. Samples of 5–8 mg encapsulated in tzero pans were quenched at a rate of 20 °C/min to −75 °C and kept under isothermal conditions for 3 min followed by heating at 5 °C/min to 160 °C and then cooling at 5 °C/min. The method was adapted from ref (17).

### 2.6 Mechanical Testing of PVDF-TrFE

Mechanical analysis of five thin films was performed using a Zwick/Roell Z010 with a 1 kN load cell, a crosshead speed of 500 mm/min, and a maximum extension of 500% in accordance with ASTM D 882.

### 2.7 Quasi-Static Measurements of the Piezoelectric Coefficient

The piezoelectric *d*_33_ coefficient of the thin films was measured by sandwiching PVDF-TrFE films between two parallel electrodes under a quasi-static oscillatory force of 250 mN at a frequency of 111 Hz using a commercial piezometer (PM300, Piezotest, London, U.K.).

### 2.8 Open-Voltage Measurement

PVDF-TrFE films were fitted to custom cell culture plates as previously described under 3 mL of cell media: Dulbecco’s modified Eagle’s medium (DMEM; Gibco) with 10% fetal bovine serum (FBS; Gibco), 1% nonessential amino acids (Sigma-Aldrich), 1% 100 mM sodium pyruvate (Sigma-Aldrich), 200 nM l-glutamate (Sigma-Aldrich), 10 mg/mL penicillin/streptavidin (Sigma-Aldrich), and 0.5% Fungizone (Thermo Fisher).^10^ Poled and unpoled films were then sputter-coated with gold to form 16-mm-diameter electrodes on both sides, ensuring that electrical shorting did not occur. The voltage generated from PVDF-TrFE films oscillated at a frequency of 1000 Hz with the Nanokick bioreactor was measured using a PowerLab 8/35 instrument (ADI Instruments) and further processed using a Labchart (ADI Instruments).

### 2.9 X-ray Photoelectron Spectroscopy (XPS)

After plasma polymerization, samples were sent to HarwellXPS (found at http://www.harwellxps.uk/), Cardiff University, and University College London for XPS spectral analysis. A Kratos SUPRA XPS spectrometer fitted with a monochromated Al Kα X-ray source (1486.69 eV; high tension = 15 kV; emission current = 15 mA) and an electron flood gun charge neutralizer for carbon, oxygen, nitrogen, and bromine and overview spectra was used to analyze each sample in three different locations at a maximum beam size of 400 μm × 800 μm. Spectral analysis and curve fitting were performed by using *CasaXPS* software.

### 2.10 Atomic Force Microscopy (AFM)

A JPK Nanowizard 4 (JPK Instruments) was used for imaging in alternating-contact mode using antimony-doped silicon cantilevers with a nominal resonant frequency of 75000 Hz and a force constant of 3 N/m (MPP-21120, Bruker). AFM scans were used to visualize topology before and after FN coating in all conditions on samples in ambient conditions. FN-coated samples were rinsed with water after FN adsorption and gently dried with a nitrogen flow. Surface area scans (*n* ≥ 3) of 5 × 5 μm^2^ (0.5 Hz) were utilized to calculate the average surface roughness. Height and phase images were acquired from each scan, and the JPK data processing software version 5 was used for image analysis.

### 2.11 Water Contact Angle (WCA)

WCA measurements were taken on PVDF-TrFE membranes before and after PEA coating by dropping 3 μL of deionized water onto the surfaces using a Theta optical tensiometer (Biolin Scientific, Stockholm, Sweden).

### 2.12 Cell Culture

Human bone marrow MSCs were purchased from Promocell and cultured in DMEM (Sigma-Aldrich) with 10% FBS (Thermo Fisher), 1% nonessential amino acids (Sigma-Aldrich), 1% 100 mM sodium pyruvate (Sigma-Aldrich), 200 nM l-glutamate (Sigma-Aldrich), and an antibiotic mixture consisting of 10 mg/mL penicillin/streptavidin (Sigma-Aldrich) and 0.5% Fungizone (Thermo Fisher). Cells were incubated in a 5% humidified CO_2_ atmosphere at 37 °C. MSCs were expanded and used up to passage 4. For cell culture on PVDF-TrFE films, cells were seeded at 2000/cm^2^ and cultured for the indicated time point. Cells were first allowed to adhere to films overnight, and then nanovibration was introduced. The medium was exchanged every 3 days. For the experiments, three technical replicates were used from one biological donor, with multiple donors used in independent experiments.

### 2.13 Nanovibration

The design of the nanovibrational bioreactor has been previously described.^19^ Custom cell culture plates (Proto Laboratories Ltd., Telford, U.K.; Figure 1A) were magnetically attached (NeoFlex Flexible Neodymium Magnetic Sheet, 3M, St. Paul, MN) to the vibration plate (dimensions, 128 mm × 176 mm). The vibration plate was secured on its underside to an array of low-profile, multilayer piezo actuators (NAC2022, Noliac A/S CTS, Kvistgård, Denmark). To power the piezoarray, a custom power supply unit was used, as detailed in a previous publication,^28^ consisting of a signal generator integrated circuit (AD9833, Analog Devices, Wilmington, MA) to provide a 1000 Hz sine wave modulation and a parallel configuration of class AB audio amplifiers (TDA7293, STMicroelectronics, Geneva, Switzerland) in order to amplify the sine-wave signal. This results in the vibration plate oscillating at an amplitude of 30 nm and a frequency of 1000 Hz.

### 2.14 Immunocytochemistry

MSCs were cultured on films for the times indicated and fixed using 4% formaldehyde for 15 min. Cells were then permeabilized with 0.5% Triton-X for 5 min and blocked using 0.5% bovine serum albumin (BSA)/PBS for 2 h at room temperature (RT). Primary antibodies were then added in 0.5% BSA/PBS: antivinculin hVIN-1 (1:200; Sigma-Aldrich); osterix (OSX; 1:200; Abcam, ab209484); osteonectin (ON; 1:200; Santa Cruz Biotechnology, sc398419). Cells were then washed 5 × 5 min with PBS/Tween 20 (PBST), and biotinylated secondary antibodies (1:50; Vector Laboratories) were added in a blocking buffer for 2 h at RT. Cells were again washed 3 × 5 min in PBST and incubated with fluorescein isothiocyanate-conjugated streptavidin (1:50; Vector Laboratories) in a blocking buffer for 30 min at RT. Nuclei were stained using VECTASHIELD mountant with 5′,6-diamidino-2-phenylindole nuclear stain (DAPI; Vector Laboratories). Samples were then mounted onto glass slides and visualized using an Axiophot microscope or an Evos Cell imaging system (Thermo Fisher). For viability analysis, at the indicated time point, independent films were stained using a LIVE/DEAD viability kit (Thermo Fisher, L3224) as per the manufacturer’s instructions. Briefly, live cells are labeled with calcein-AM and dead cells with ethidium homodimer-1; the labeled cells were then visualized using the onstage incubator on the Evos Cell imaging system (Thermo Fisher). The total number of cells detected in both channels was then counted, and the percentage of cells in each channel was calculated to give the percent viability. All image analyses were carried out using *ImageJ* software (National Institutes of Health).

### 2.15 FN Adsorption Assays

To quantify the amount of FN adsorbed onto PVDF-TrfE–PEA, films were coated with a 20 μg/mL FN/PBS solution for 1 h, aspirate was collected, and FN was quantified using a Pierce BCA Protein Assay Kit (Thermo Fisher Scientific U.K.) as per the manufacturer’s instructions. Quantitative immunofluorescence assays were carried out using a LI-COR in-cell western platform. FN (20 μg/mL) was adsorbed as previously described, and samples were washed with PBS, blocked with 1% milk protein in PBS, and incubated with primary antibodies for total FN (polyclonal rabbit, Sigma-Aldrich), HFN7.1 (monoclonal mouse, Developmental Studies Hybridoma Bank, Iowa City, IA), and P5F3 (monoclonal mouse, Santa Cruz Biotechnology, sc-18827) for 2 h. Substrates were washed five times with 0.5% PBST, followed by incubation o/n at 4 °C with LI-COR secondary antibodies (IRDye 800CW/700CW antirabbit/mouse secondary antibody, LI-COR Biosciences U.K.). The samples were washed five times with PBST, followed by a final wash in PBS and drying before imaging on a LI-COR Sa Odyssey scanner.

### 2.16 Statistics

All statistical analysis was performed using *GraphPad Prism* software (version 8.0.0; GraphPad Software Inc., San Diego, CA).

## 3 Results and Discussion

### 3.1 Generation of Unpoled and Poled PVDF-TrFE Films

PVDF-TrFE provides an interesting building block to utilize piezoelectric currents within biomedical approaches for bone regeneration.^13,14,17^Table 1 shows the thermal and mechanical properties of PVDF-TrFE as-received and PVDF-TrFE cast films. During the cooling and heating DSC cycles, both PVDF-TrFE formulations showed two clear peaks. The first and second peaks of as-received PVDF-TrFE were observed at 118 °C (Curie temperature) and 138 °C (melt temperature) and then shifted to 199 and 138 °C in cast PVDF-TrFE films. Conversely, these two peaks were observed at 120 and 73 °C for both PVDF-TrFE formulations during the cooling cycle. A greater enthalpy of annealed films during the melt phase suggests higher crystallinity, indicating a higher β-phase content than that reported in a previously work.^17^ Hence, 120 °C was chosen as the annealing temperature for subsequent β-phase enhancement and increased crystallinity. The crystallinity and rate of crystallization in PVDF-TrFE are further outlined in Figures S1 and S2. PVDF-TrFE films were annealed at 120 °C and poled under a voltage of 100 V/μm for 5 min. Poled films showed a *d*_33_ coefficient ranging from −10 to −12 pC/N, whereas annealed but unpoled films showed a *d*_33_ coefficient ranging from 0 to −0.3 pC/N (see methods; data not shown).

**Table 1:** Thermal and Mechanical Properties of PVDF-TrFE

						temperature (°C)	
material	Young’s modulus (MPa)	modulus of resilience (MPa)	modulus of toughness (MPa)	crystallization temp (°C)	heat of enthalpy (J/g)	heat of crystallization cooling cycle	heat of crystallization – cooling cycle
PVDF-TrFE (as-received)				120 ± 0.16	32.34 ± 1.03	25.07 ± 0.93	18.81 ± 0.83
PVDF-TrFE films (casted and annealed)	816.2 ± 23	72.72 ± 7.2	3417.0 ± 202	120 ± 0.09	34.82 ± 1.03	27.36 ± 0.83	16.47 ± 1.83

PVDF-TrFE films were then mounted on custom cell culture plates containing a volcano-like shape in each well that supports taut fixation of the films for cell culture (Figure 1A). These plates are then magnetically attached to the NK bioreactor plate to mechanically stimulate PVDF-TrFE. Scanning interferometry was used to measure the displacement over a range of frequencies (Figure S3). The measurements are summarized in Tables S1 and S2, and the average *z*-displacement across the NK bioreactor plate and PVDF-TrFE films mounted on the cultureware is shown in Figure 1B. This demonstrates that the vibration from the bioreactor is successfully transmitted to the films in each of the wells of the cultureware, and at frequencies between 700 and 2200 Hz, there is some amplification of the vibration. A frequency of 1 kHz was chosen because it leads to displacements within the range previously described for the osteogenesis of MSCs.^19^ The average *z*-displacement was 87 ± 64.26 nm (Table S1). The large variation was attributed to inconsistencies between the individual wells of the culture plate due to fixing of the film to the well plate. As such, the method of attaching the films to the volcano plate was optimized for further experiments to minimize variation. Next, the voltage generated by the poled and unpoled PVDF-TrFE films mounted on volcano culture plates subjected to 1 kHz vibration was measured. Figure 1C shows that the *V*_pp_ values for unpoled and poled films were measured at 0.6 ± 0.1 and 1.6 ± 0.03 mV, respectively.

### 3.2 Surface Characterization of pPEA-Treated PVDF-TrFE Films

**Figure 2:**
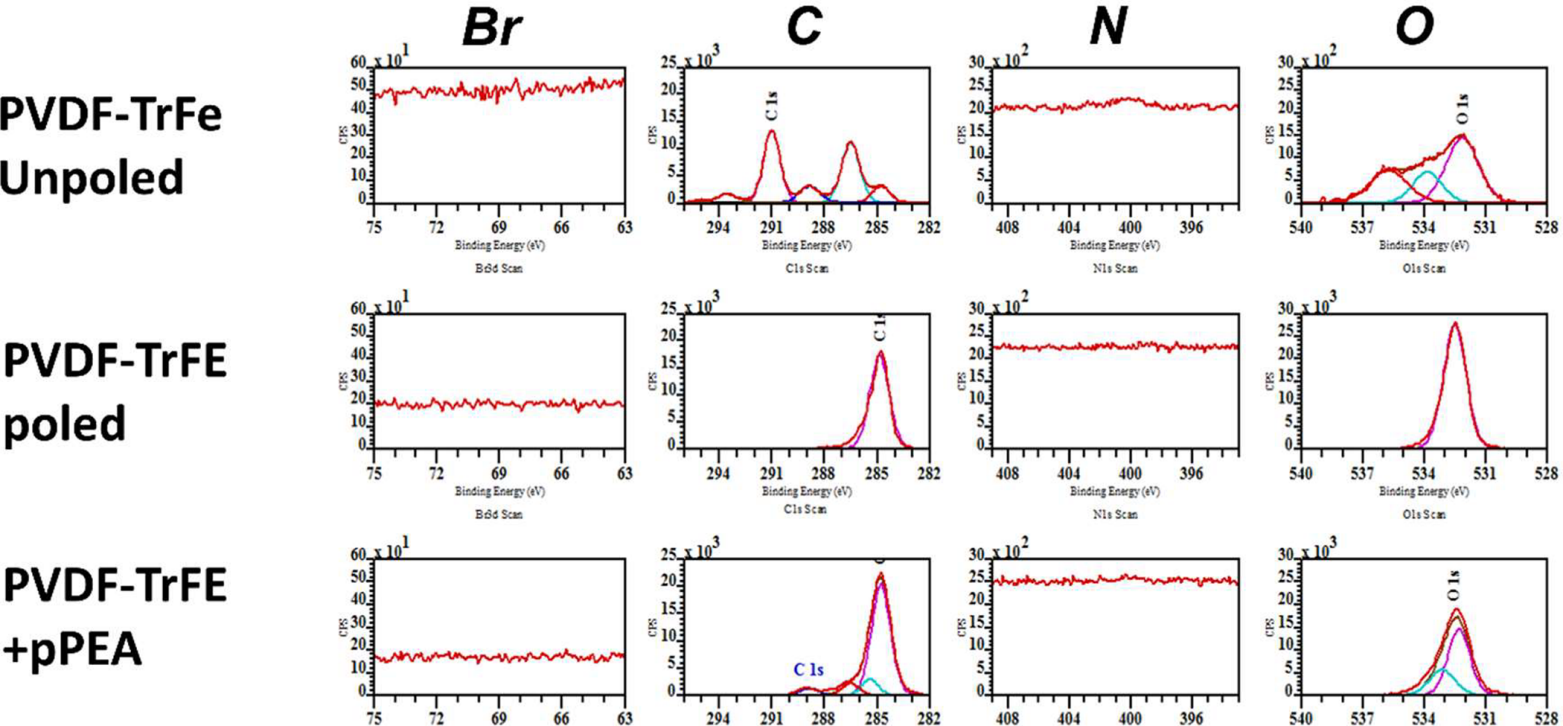
Chemical composition of PVDF-TrFE surfaces. The chemical composition and surface chemical binding characteristics of both poled and unpoled PVDF-TrFE before and after plasma polymerization of PEA is shown. The C1 carbon (C), N1 nitrogen (N), and O1 oxygen (O) core-level spectra of PVDF-TrFE films through surface modification processes were taken by XPS analysis. Each spectrum represents the binding environment of the given element within the top 10 nm; each peak was fitted and characterized to identify the presence of an element of binding conformation, where no peak signifies the absence of that element within the given condition. The spectra for both films were observed to be identical postpolymerization, representing the carbon and oxygen spectra of pPEA.

Poled and unpoled PVDF-TrFE films were next coated with PEA. PEA coating on glass and tissue culture plastic has been shown to drive spontaneous fibrillogenesis of adsorbed FN, which can then be used to efficiently present GFs in the solid phase to cells.^24−27^ Previously, several methods for PEA coating have been investigated, such as plasma polymerization,^25,29^ spin coating,^30,24^ and surface-initiated atomic transfer radical polymerization (SI-ATRP).^31^ We predicted that spin coating PEA onto PVDF-TrFE films would be nonfacile, and spin coating typically yields PEA coatings of ≈1 μm thickness, which may render the poling state of the films redundant.^24,25^ SI-ATRP of PEA requires an abundance of chemical processing; as such, it was speculated that this may impact the charge induced in the PVDF-TrFE films during poling.^31^ Because plasma polymerization does not require chemical processing and is able to produce PEA coatings in the less than tens of nanometer range, we chose to investigate this method. Therefore, to create a nanoscale layer of PEA on PVDF-TrFE films, we plasma-polymerized PEA (pPEA) using our previously described system.^22,25,27^ To validate the incorporation of PEA onto poled and unpoled PVDF-TrFE films, we carried out XPS analysis. The spectra of pristine unpoled (−pole) PVDF-TrFE samples were identical with those presented in the literature,^17,32^ representing chemical binding on the top 10 nm associated with the theoretical chemical composition (Figure 2). Poled (+pole) PVDF-TrFE was observed to possess a severely altered surface chemical composition compared to that of −pole PVDF-TrFE, presenting a single peak in both the carbon and oxygen spectra. These single C–C carbon (285 eV) and organic C–O oxygen (532 eV) peaks, in the carbon and oxygen spectra, respectively, can be attributed to the poling process. After surface modification with pPEA, both the −pole and +pole spectra were identical and matched our previous XPS analysis for pPEA coating.^22,25^

**Figure 3:**
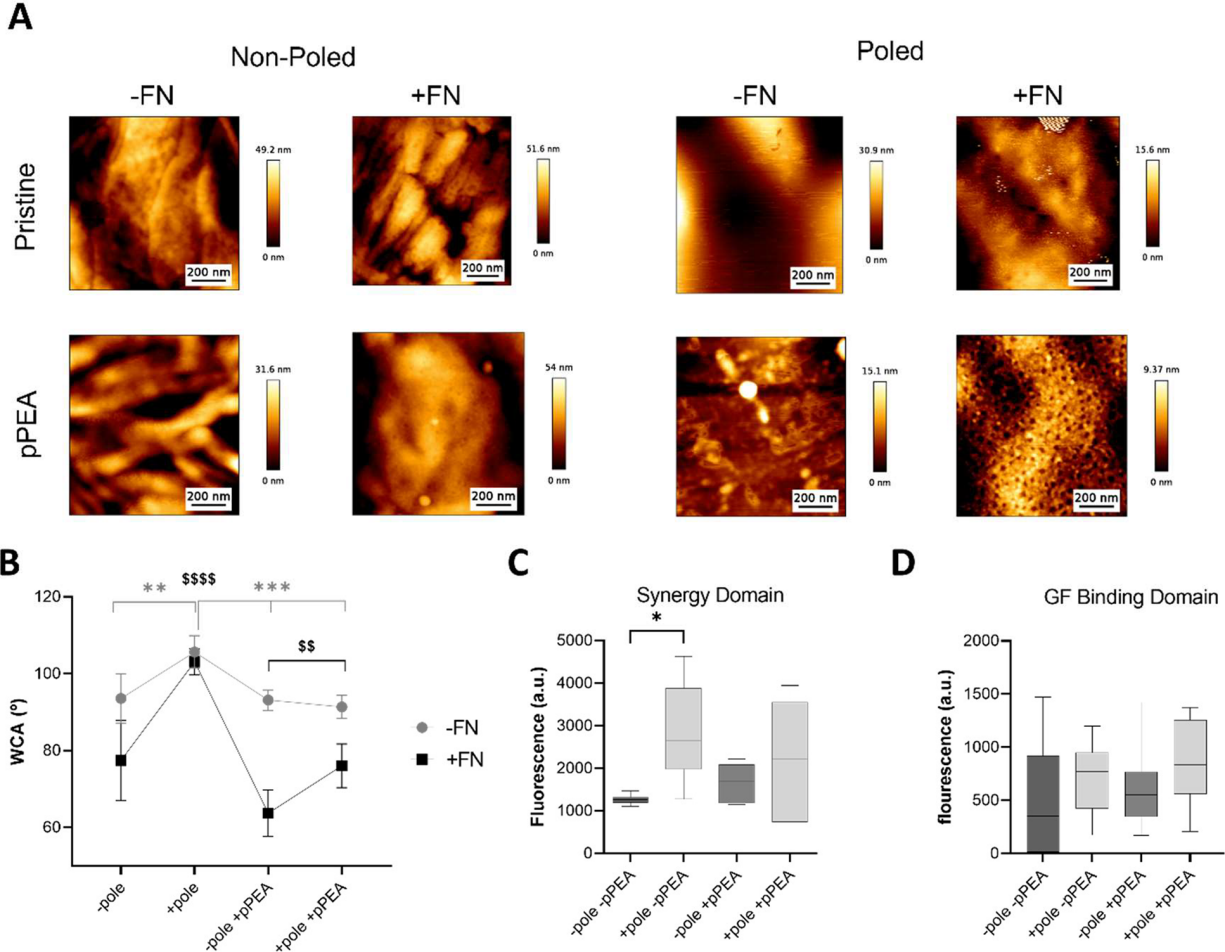
Surface characterization of PVDF-TrFE films after plasma PEA modification and FN treatment. (A) Surface topography of both poled and nonpoled films before and after pPEA and FN treatment shown via AFM height scans. FN nanonetworks can be visually observed on poled pPEA conditions. (B) Static WCA analysis of PVDF-TrFE films with and without pPEA and FN treatment. pPEA and FN treatment significantly reduces hydrophobicity; *n* = 3 material replicates with repeated measurements. */gray indicates −FN; $/black indicates +FN. (C) Synergy integrin binding domain and (D) GF binding domain availability showing a distinct trend of upregulation in the availability of both domains in poled conditions. Graphs show mean ± standard deviation statistics by one-way ANOVA with Tukey multiple comparisons: *, *p* < 0.05; **/$$, *p* < 0.005; ***, *p* < 0.001; $$$$, *p* < 0.0001; *n* = 3 material replicates.

Next, to characterize the ability of pPEA to drive efficient FN fibrillogenesis, films were characterized via AFM to establish pristine surface (−pPEA) topography and to discern the physical and functional impact of pPEA coating and FN treatment on PVDF-TrFE. Visually, both −pole and +pole pristine (−pPEA) surfaces were observed to have similarly uniform topographies. pPEA coating led to distinct features that could be observed on these surfaces by AFM (Figure 3A), where the −pole surfaces resemble that of overlapping strands and the +pole surfaces seem to produce a more uniform layer with sporadic islands of height made potentially exclusively of clustered pPEA. However, this variation was not observed to alter the average root-mean-square (RMS) roughness (Figure S4A). Films were then treated with FN, and no distinct secondary structural alterations were observed on any surfaces, with the exception of poled PVDF-TrFE + pPEA (+pole + pPEA + FN) where FN nanonetworks were present (Figure 3A). The presence of very tightly bound, smaller nanonetworks was speculated to be present on nonpoled + pPEA samples, represented by very small holes, which is typical on plasma-polymerized samples.^22^ However, due to the size of these features, the required resolution could not be obtained using AFM. In general, no significant alterations in RMS roughness were observed between treatment conditions with the exception of the poled PVDF-TrFE + pPEA treated with FN (+pole + pPEA + FN) (Figure S4A). This further confirmed the presence of a flat FN nanonetwork topography on these surfaces because FN is able to overlay the features present before protein treatment and present a flatter monolayer of fibular networks.^22,24,25,31^

The surface chemistry and related hydrophilicity was evaluated utilizing the WCA both before and after FN treatment (Figure 3B). Poled pristine PVDF-TrFE (+pole – pPEA + FN) was observed to be significantly more hydrophobic compared to the nonpoled control (−pole – pPEA + FN). However, after pPEA coating (+pole + pPEA + FN), the hydrophilicity was increased to levels comparable to those of both pristine and pPEA nonpoled samples (−pole – pPEA + FN/–pole + pPEA + FN), suggesting a uniform pPEA coating that masks the hydrophobic nature of the poling. Similar characteristics were observed after FN treatment (Figure 3B). However, in general, FN-treated samples were observed to have lower contact angles; because FN is a hydrophilic protein, this was expected.^31^ This suggests that the observed hydrophobicity of PVDF-TrFE + pole – pPEA may affect FN adsorption to the material surface. Interestingly, −pole + pPEA surfaces had a significantly lower contact angle and, therefore, higher hydrophilicity than any other conditions after FN treatment. This may support the observation of small tightly bound nanonetworks observed in the AFM analysis (Figure 3A).

To further characterize the protein interactions on the pPEA-treated PVDF-TrFE films, the total surface density and the availability of specific cryptic binding domains of FN were measured. No significant variation was observed in the total density of FN on any surfaces; this is in line with previous investigations on surfaces after PEA modification^24,30,31^ (Figure S4B). Both the synergy (PHSRN) and GF binding domains show a similar trend, in which +pole ± pPEA surfaces present higher availability of these binding domains (Figure 3C,D). While this increased adhesion and GF binding domain availability is in line with the nanonetworks observed in Figure 3A on PVDF-TrFE + pole + pPEA surfaces, it was not expected on the condition −pPEA. PVDF-TrFE + pole −pPEA was observed to present the highest degree of hydrophobicity that was retained even after FN treatment; interestingly, this resulted in a relative increase in the presentation of cryptic FN binding domains.^33^ This may indicate that the charged nature of the poled PVDF-TrFE is able to induce some degree of restructuring of the secondary structure of the FN. We could postulate that this interaction may synergize with the pPEA coating and present FN nanonetworks on the AFM, whereas the −pole surfaces would present dense pPEA-driven FN nanonetworks that are difficult to visualize with AFM.^25^ Surface poling and charge have been previously reported to influence the FN secondary structure.^34^ Here, poled PVDF-TrFE samples were measured to have significantly higher hydrophobicity than the nonpoled samples, both before and after pPEA treatment. Alongside this hydrophobicity, +pole PVDF-TrFE had a piezoelectric *d*_33_ coefficient of ∼30 pC/N (data not shown), suggesting that the surface electrostatic characteristics of the films themselves may have an influence in FN fibrillogenesis.^10^ By characterizing the surface of the PVDF-TrFE films after pPEA treatment, we observed distinct variations in both polymer coating and FN network formation on poled and nonpoled polymers, resulting in the formation of FN nanonetworks and enhanced presentation of the cryptic FN binding domains.

### 3.3 pPEA Coating of PVDF-TrFE Increasing the MSC Viability and Focal Adhesion (FA) Formation

**Figure 4:**
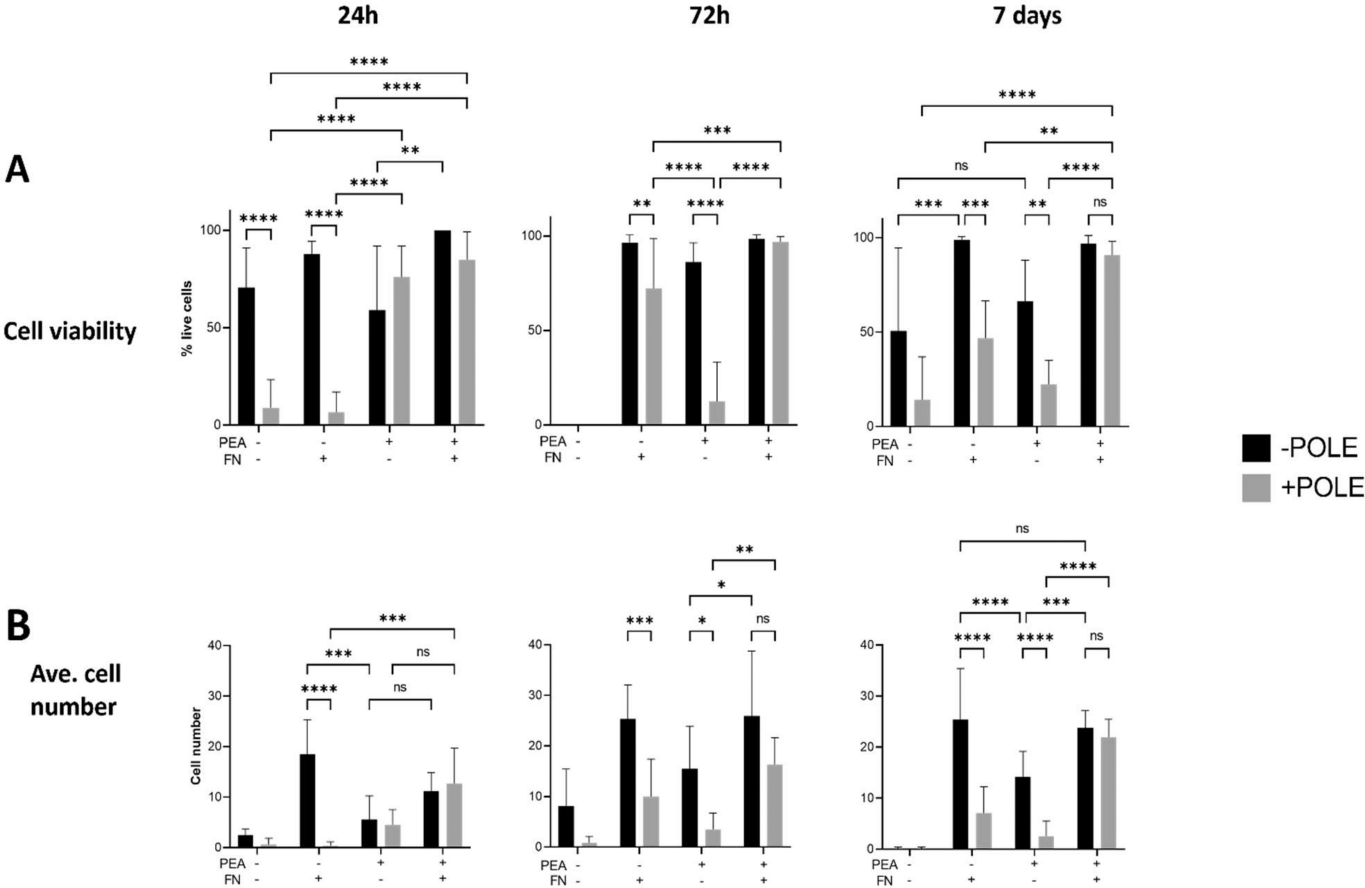
Characterization of the MSC viability and number on surface-modified PVDF-TrFE films up to 7 days. Graphs show (A) the cellular viability, measured via live/dead analysis, and (B) the average cell number (per frame) on unpoled (−POLE) and poled (+POLE) PVDF-TrFE films coated with or without pPEA and 20 μg/mL FN at 24 h, 72 h, and 7 days. Graphs show mean and standard deviation statistics by two-way ANOVA with multiple comparisons, *n* ≥ 15: *, *p* < 0.05; **, *p* < 0.01; ***, *p* < 0.001; ****, *p* < 0.0001; *n* = 3 material replicates from one biological donor.

Having established the surface characteristics and successful coating of pPEA onto the PVDF-TrFE films using plasma polymerization, cellular interactions were investigated to ascertain whether pPEA-driven FN fibrillogenesis was able to enhance the cellular viability on PVDF-TrFE films. MSCs were cultured on films for up to 7 days. PVDF-TrFE – pole – pPEA – FN displayed an initial high cell viability (24 h), which decreased at 72 h, with no attached cells detected by day 7 (Figure 4A). Quantification of the cell number (per frame) indicates that this initially observed high viability is due to a low number of cells attaching (Figure 4B). The cell viability and number were observed to be high on all other −pole films, including the −pole + pPEA – FN condition; typically pPEA without FN leads to low cell viability and adhesion, which is observed on +pole + pPEA – FN films at all time points (Figure 4A,B). Poling (+pole) of PVDF-TrFE films led to significant reductions in the cell viability and number. However, when +pole PVDF-TrFE was coated with pPEA and FN (PVDF-TrFE + pole + pPEA + FN), the viability significantly increased (Figure 4A). This suggests that the inclusion of pPEA and FN in poled films may enhance the use of this system for potential bioengineering applications, corroborating the increased surface hydropholicity observed on +pole PVDF-TrFE when coated with pPEA and FN in Figure 3.

**Figure 5:**
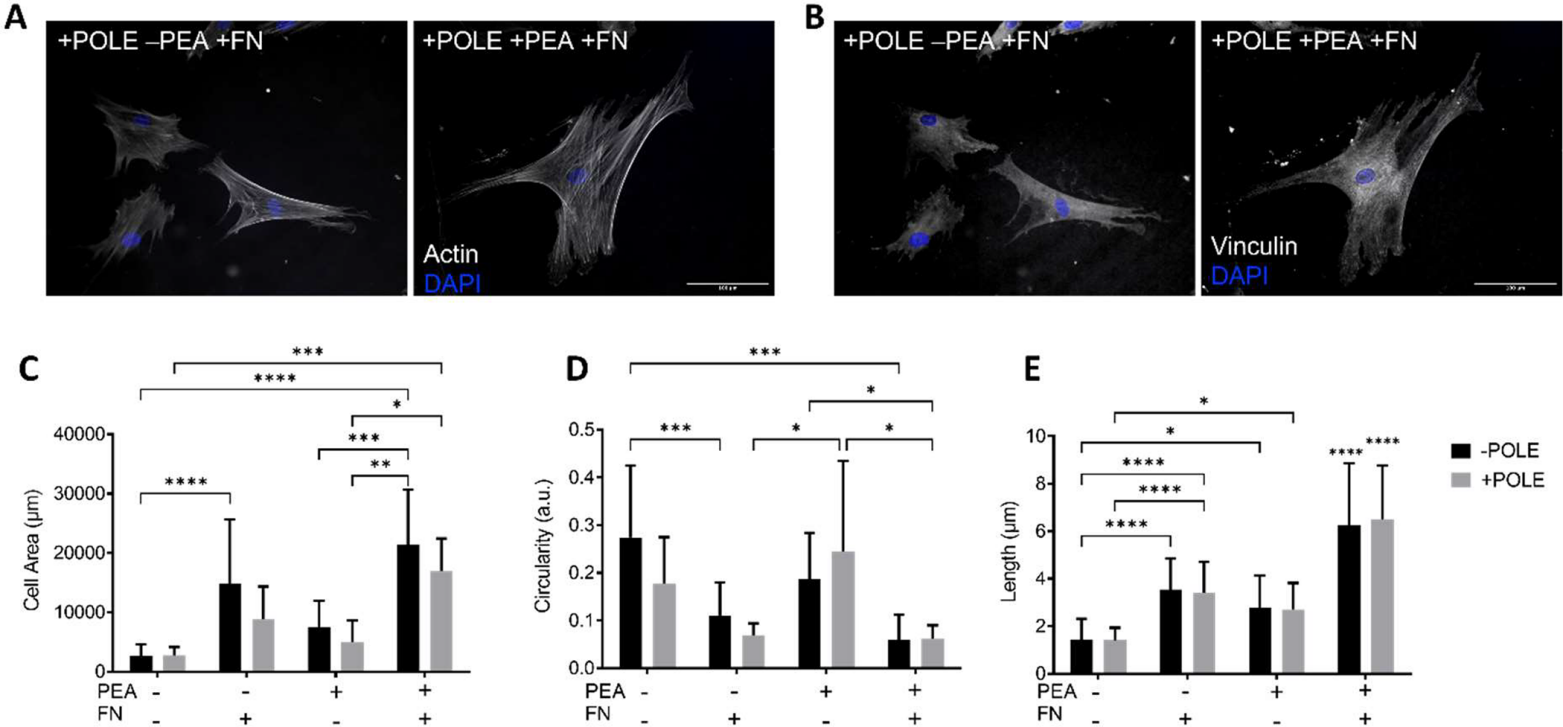
Initial cellular adhesion. FA analysis of the MSC surface interactions onto −pole and +pole PVDF-TrFE films coated with pPEA and FN after 4 h. Representative images of poled PVDF-TrFE films + FN ± pPEA treatment: (A) actin (phalloidin); (B) FAs (vinculin). (C) Surface area and (D) cell circulatory measured via phalloidin staining of MSCs. It is indicated that +pPEA + FN coated films lead to an increase in cell spreading and a decrease in cell circularity in both ±pole conditions. (E) Average FA length measured from vinculin staining. +pPEA + FN led to a significant increase in the average length of FA in ±pole PVDF-TrFE films. Graphs show mean and standard deviation statistics by two-way ANOVA with multiple comparisons:. *, *p* < 0.05; **, *p* < 0.01; ***, *p* < 0.001; ****, *p* < 0.0001; *n* = 3 material replicates from one biological donor; ≥15 cells measured per condition.

To further characterize the cellular interactions on surface-modified PVDF-TrFE films, FAs were analyzed. MSCs were cultured on films for 4 h, and FA formation was assessed by vinculin staining (Figure 5B). Figure 5B shows that cells were attached on both +pPEA and −pPEA + pole films. However, without pPEA, cells are smaller and less spread out. MSCs cultured on +pPEA + FN films, both +poled and −poled, showed a significantly increased total surface area (Figure 5C) compared to all other conditions (PVDF-TrFE ± pole + pPEA + FN). Additionally, a significantly lower cellular circularity was observed on films −FN (Figure 5D). The FA length was observed not to be impacted by the poling state for any condition (Figure 5E). Pristine samples without FN were observed to present the lowest average FA length (±pole – pPEA – FN), while conditions with pPEA or FN showed slightly higher FA lengths (Figure 5E). However, the combination of pPEA and FN led to significantly longer FA formation independent of poling (Figure 5E). These results corroborate with the cellular characterization data presented in Figure 4 because −pPEA – FN films did not facilitate long-term cellular culture, whereas +pPEA + FN conditions were able to. In summary, independent of poling, +pPEA + FN leads to enhanced cell spreading, a decrease in the cell circularity, and enhanced FA formation in MSCs cultured on PVDF-TrFE membranes, which are all typical of the early osteogenic commitment of MSCs.^35^

### 3.4 Nanovibration Enhancing the Adhesion and Early Osteogenic Commitment of MSCs on Poled and Unpoled PVDF-TrFE + pPEA + FN

Nanovibration, or nanokicking (NK), of MSCs has been shown to promote the commitment to osteogenesis.^19,20^ Having established that coating PVDF-TrFE with pPEA and FN leads to increased cell viability (Figure 3) and FA formation of MSCs (Figure 4), NK was incorporated into the system with the aim of driving the osteogenic commitment. To investigate the effect of mechanically induced piezoelectric stimulation on MSCs on PVDF-TrFE + pPEA + FN films, we mounted ±pole PVDF-TrFE + pPEA + FN films onto custom-made volcano tissue culture plates (Figure 1A), resulting in an amplitude of 87.1 nm at 1 kHz nanokicking^19,20,28^ (Figures 1B and S3).

**Figure 6:**
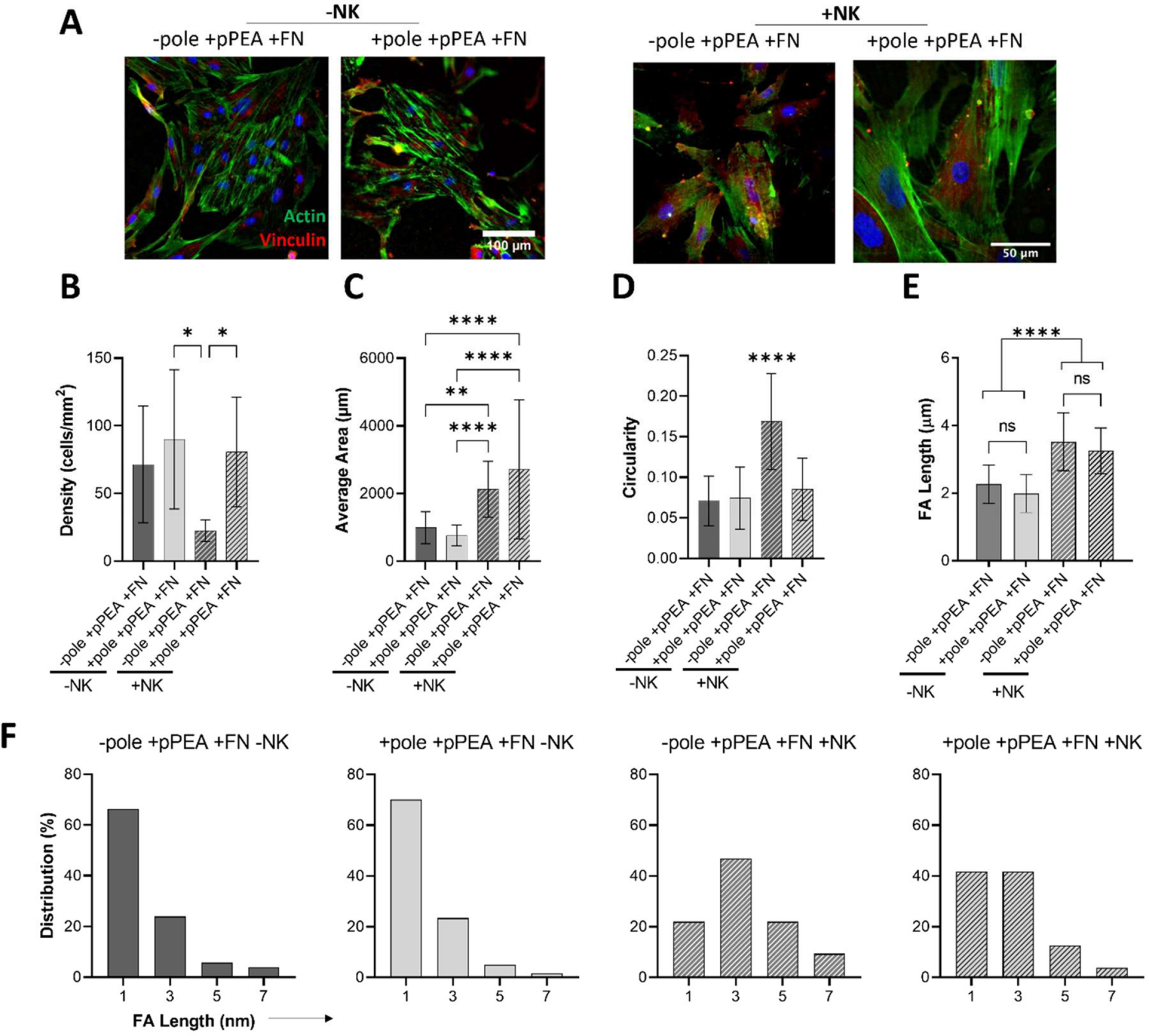
Nanokicking of PVDF-TrFE–pPEA enhancing MSC FA formation. Nanokicking the surface-modified PVDF-TrFE films was characterized to evaluate the cell and adhesion morphology. (A) Representative images of poled and unpoled films with and without nanokicking: blue, DAPI; green, phallopidin; red, vinculin. (B) Calculated cellular density on each condition. (C) Average cell area. (D) Cellular circularity. (E) Average FA length. (F) Calculated frequency distribution of FAs. Graphs show mean and standard deviation statistics by one-way ANOVA with Tukey multiple comparisons, *n* = 3 material replicates, from one biological donor; ≥15 cells measured per replicate.

MSCs were cultured on PVDF-TrFE ± pPEA ± FN ± NK for 3 days. Representative images of poled and nonpoled + pPEA + FN films (Figure 6A) show that cells on +NK films spread significantly more (both ±pole) compared to static cultures (Figure 6C). However, significantly lower cellular density and high circularity were measured on −pole + NK (Figure 6B), compared to +pole conditions, suggesting that poling is advantageous to cell attachment upon mechanical stimulation. Vinculin staining was then used to assess FA formation (Figure S5). The average FA length was significantly higher in response to mechanical stimulation (±pole + NK) compared to that of static films (Figure 5E). Histograms indicating the size distribution of FAs further show that in static (−NK) films the highest percentage of measured FAs is smaller, immature ≤1 μm adhesions (Figure 5F), whereas nanovibration (+NK) of films, regardless of the poling state, leads to a ≥2-fold increase in the percentage of larger 3–5 μm adhesions, suggestive of larger, mature adhesion formation. Together, these data suggest that poled films + pPEA + FN better support cell attachment, leading to enhanced cell spreading and mature FA formation in response to nanovibrational stimulation.

**Figure 7:**
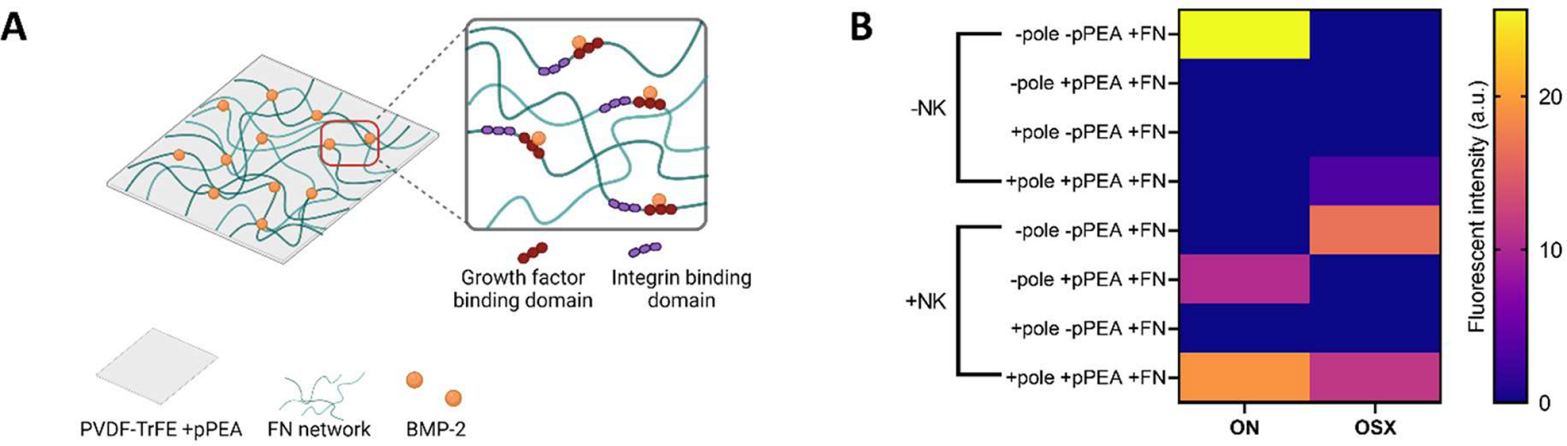
Osteogenic potential of PVDF-TrFE + pPEA + FN with low-dose BMP-2. (A) Schematic illustrating PVDF-TrFE films coated with pPEA to drive the network formation of FN. FN unfolding reveals cryptic GF and integrin binding domains; thus, low doses of GFs such as BMP-2 can be adsorbed and presented in the solid phase.^24,25^ Created using Biorender.com. (B) MSCs were cultured on PVDF-TrFE films ± pole ± pPEA + FN + BMP-2 with or without nanovibration (NK; nanokicking) for 14 days. Expression of osteogenic markers ON and OSX was analyzed by fluorescent microscopy. An increase in both the ON and OSX expressions was observed in +pole + pPEA + FN + NK films. *n* = 3 material replicates from one biological donor.

Finally, to evaluate the osteogenic potential of this system, the osteogenic GF BMP-2 was adsorbed to PVDF-TrFE films + pPEA + FN to support a low-dose (100 ng/mL), solid-phase presentation to MSCs^24,25^ (Figure 7A). Cells were cultured for 14 days, and Figure 7B shows the relative fluorescent intensity of early osteogenic markers ON and OSX. Expression of both ON and OSX was increased only on +pole + pPEA + FN films that were subjected to nanomechanical stimulation. This was increased relative to static +pole + pPEA + FN films, suggesting that nanovibration could enhance the osteogenic commitment on these surface-modified films, which is in agreement with nanovibration enhancing FA formation and initial cellular spreading on this condition (Figure 6).

## 4 Conclusions

Taken together, the data suggest that +pole PVDF responds to mechanical stimulation, yet +pole pristine PVDF-TrFE does not support cell–material interactions. Enhancing adhesive interactions onto the film surfaces through surface modification with +pPEA + FN allowed for the incorporation of low-amplitude, high-frequency mechanical stimulation, nanokicking to the system. The nature of the piezoelectric films requires a degree of deformation to fully optimize the charge induced from poling. PVDF-TrFE films have been shown to synergize with 1 Hz, 30 nm amplitude NK to produce ∼36 pC/N charge and a modified amplitude of 87.1 nm.^10^ That nanovibrational stimulation appeared to promote the expression of early osteogenic markers suggests that nanoscale coating techniques, such as pPEA and FN, which can recreate physiologically relevant ECMs on materials, have the potential to increase the bioactivity of smart materials such as PVDF-TrFE.

The NK bioreactor was previously demonstrated to robustly promote osteogenesis of MSCs for potential clinical applications;^19^ however, the long culture time (>28 days) hinders potential clinical translation. Therefore, investigating strategies to optimize cell production from the bioreactor is highly beneficial. PVDF-TrFE is a versatile material; here we have demonstrated that inclusion of the piezoelectric stimuli in the NK system is beneficial for MSC adhesion and early osteogenic commitment. pPEA + FN coatings enhance the cellular viability and adhesion on PVDF-TrFE, and data suggest that, with the inclusion of BMP-2, the system could further enhance the osteogenesis of MSCs. Future work will further investigate the inclusion of BMP-2 to the PVDF-TrFE system and continue to investigate this systems osteogenic potential. This novel system presents multifaceted physicochemical osteoinductive cues to MSC populations through the application of nanomechanically induced electric fields. The facile nature of PVDF-TrFE fabrication alongside plasma polymerization of PEA lends well to scaling up of the system, where, e.g., PVDF-TrFE microcarriers coated with pPEA, FN, and BMP-2 could be envisioned and cultured in, e.g., spinner flasks on the NK bioreactor.

## Supporting Information Available

Additional experimental details including representative DSC curves and Avamari plots for as-received and casted PVDF-TrFE, schematic of scanning interferometry measurement grids on a bioreactor setup, roughness and total protein adsorption data, representative immunofluorescence microscopy images for FA analysis, and average displacement measurements from laser interferometry (PDF)
