# Supplementary material for: Surface-Modified Piezoelectric Copolymer Poly(vinylidene fluoride–trifluoroethylene) Supporting Physiological Extracellular Matrixes to Enhance Mesenchymal Stem Cell Adhesion for Nanoscale Mechanical Stimulation
Source: ACS Appl Mater Interfaces. 2023 Sep 18;15(44):50652–62. doi: 10.1021/acsami.3c05128 (PMC10636716; doi:10.1021/acsami.3c05128)
Supplement: Supplementary file 1 — am3c05128_si_001.pdf [file am3c05128_si_001.pdf]

Supporting Information:

Surface modified piezoelectric co-polymer poly (vinylidene fluoride-trifluoroethylene) supports physiological extracellular matrices to enhance mesenchymal stem cell adhesion for nanoscale mechanical stimulation

Hannah Donnelly[1†*] and Mark R Sprott[1*] , Anup Poudel[2], Paul Campsie[3], Peter Childs[3], Stuart Reid[3], Manus Biggs[2], Manuel Salmerón-Sánchez[1], Matthew J Dalby[1†].

[1] Centre for the Cellular Microenvironment, University of Glasgow, Glasgow G12 8QQ, United Kingdom.
[2] Centre for Research in Medical Devices (CÚRAM), National University of Ireland Galway, Galway H91W2TY, Ireland.
[3] SUPA Department of Biomedical Engineering, University of Strathclyde, Glasgow G1 1QE, United Kingdom.

*These authors contributed equally to this work.
†Corresponding authors: Hannah.Donnelly@glasgow.ac.uk; Matthew.Dalby@glasgow.ac.uk

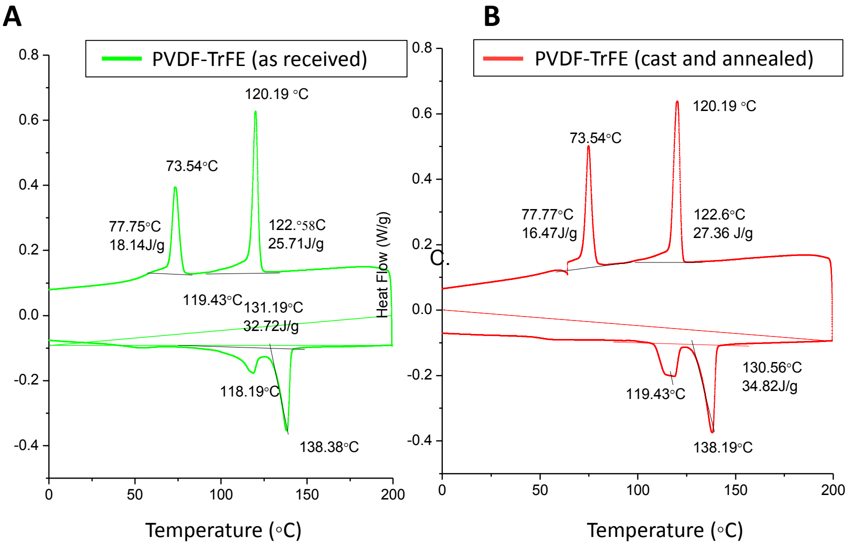

Figure S1: Representative DSC curves (heating and cooling 5˚C/min) of (A) as received PVDF -TrFE and (B) cast annealed PVDF -TrFE films. PVDF-TrFE showed two clear peaks during both the heating and cooling cycles. The first and second peak of as received PVDF-TrFE were observed at 118˚C (curie temperature) and 138˚C (melt temperature) respectively. These peaks were shifted to 119˚C and 138˚C in cast annealed PVDF-TrFE films. Similarly, the two peaks were observed at 120˚C and 73˚C for both PVDF-TrFE formulations during the cooling cycle.

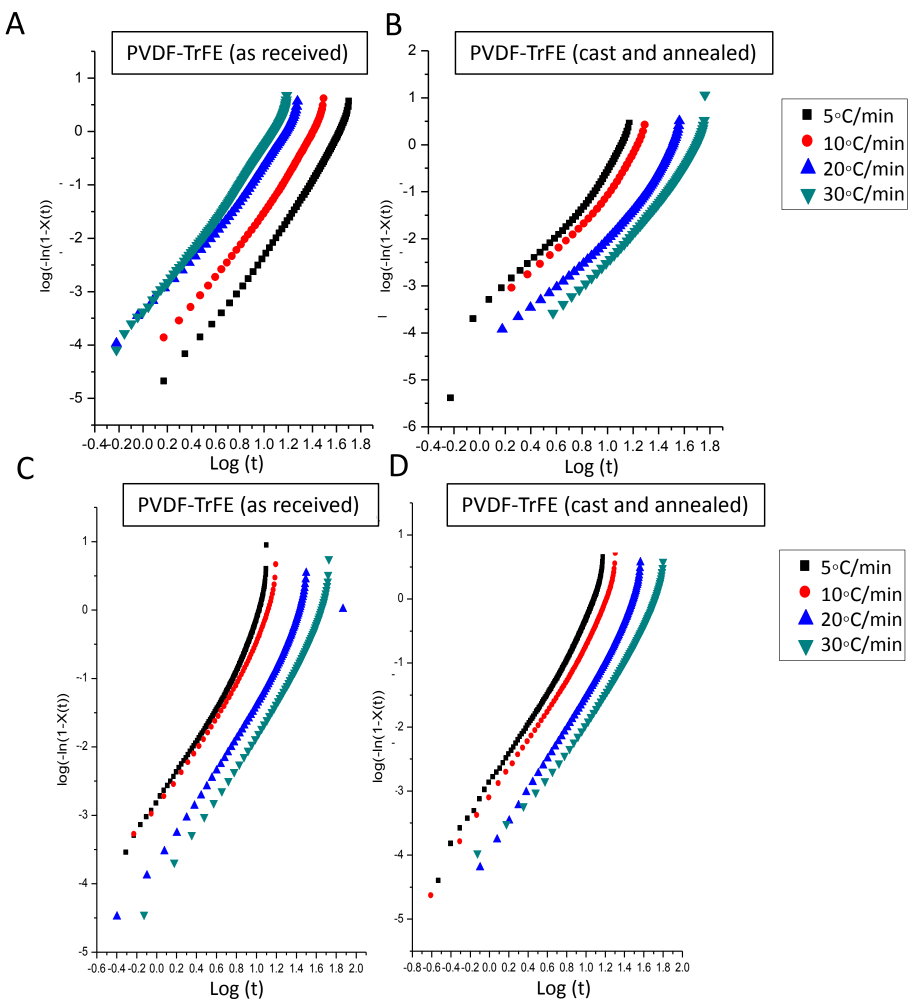

Figure S2: Avimari plots of the initial peak (A) of as received PVDF -TrFE and (B) cast annealed PVDF -TrFE films. And the secondary peak (C) of as received PVDF -TrFE and (D) cast annealed PVDF -TrFE films. crystal formation in cast annealed PVDF-TrFE films show a higher t1/2 indicating the presence of larger and more numerous crystal relative to as received PVDF -TrFE.

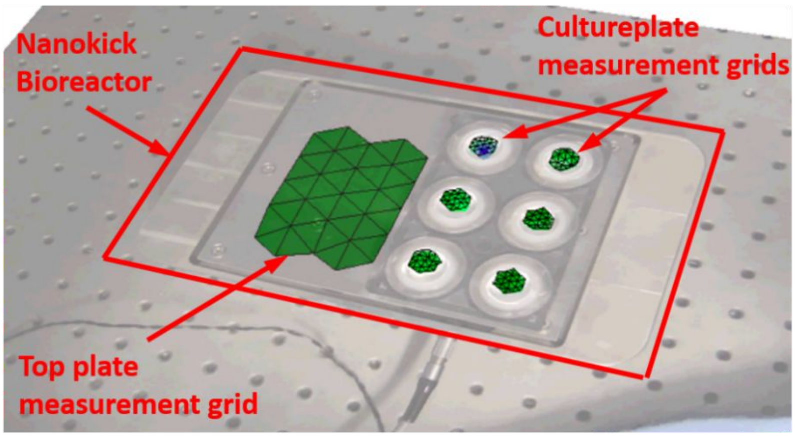

Figure S3: Scanning interferometry measurement grids overlayed on an image of the bioreactor and cultureware.

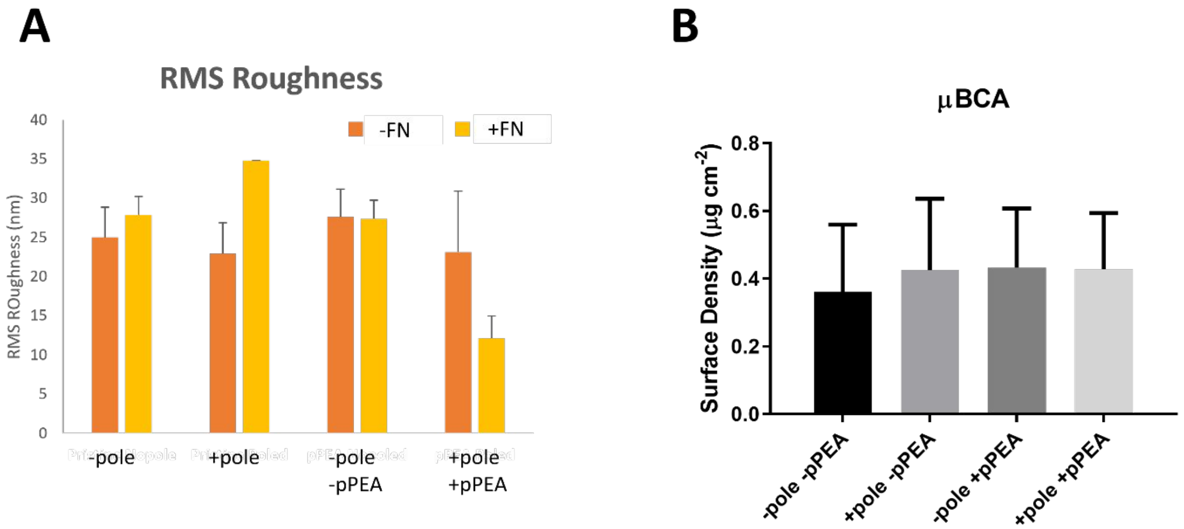

Figure S4. (A) Root mean squared (RMS) roughness analysis taken from 5 × 5 µm^2 height AFM scans. Showing no significant changes in surface roughness due to poling, pPEA or FN treatment. **(B)** Surface protein density of FN, measured via µBCA analysis. Surface densities of FN adsorbed onto each condition was uniform and there were no significant differences. Graphs show mean ±SD, n = 3 material replicates.

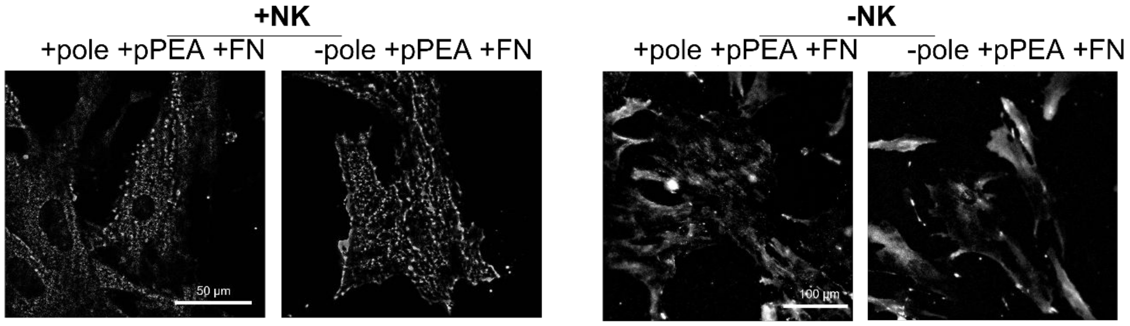

Figure S5. Binary fluorescent microscopy images of vinculin stained samples used to calculate focal adhesion lengths.

Table S1: Average displacement and standard deviation of measurement scans on the PVDF-TrFE films attached to volcano cultureware at various fixed frequencies

N Points	Voltage Amplitude from DAQ (V)	Frequency (Hz)	Average Displacement (nm)	Standard Deviation (nm)	Standard Deviation (%)
114	0.3	200	26.20	1.56	5.94
114	0.3	300	28.69	1.60	5.57
114	0.3	500	30.48	1.36	4.45
114	0.3	700	34.67	4.25	12.25
114	0.3	1000	87.10	64.26	73.78
114	0.26	1500	53.53	29.69	55.47
114	0.18	2000	100.52	117.16	116.55
114	0.08	2200	87.0	70.59	81.14

Table S2: Average displacement and standard deviation of measurement scans on the Nanokick Bioreactor top plate at various fixed frequencies with volcano cultureware attached

N Points	Voltage Amplitude from DAQ (V)	Frequency (Hz)	Average Displacement (nm)	Standard Deviation (nm)	Standard Deviation (%)
27	0.3	200	26.38	0.92	3.49
27	0.3	300	28.05	0.65	2.31
27	0.3	500	28.93	0.50	1.72
27	0.3	700	29.49	0.47	1.60
27	0.3	1000	30.11	0.58	1.92
27	0.26	1500	27.69	1.60	5.79
27	0.18	2000	24.74	7.67	31.01
27	0.08	2200	26.21	15.97	60.94
